# Temperament and the Experience of Tension and Self-Injurious Behaviour in Adolescents—The Mediating Role of Maladaptive Perfectionism

**DOI:** 10.3390/brainsci14111140

**Published:** 2024-11-14

**Authors:** Magdalena Chęć, Sylwia Michałowska, Alicja Gnych-Pietrzak, Albina Rybarska, Klaudia Strochalska

**Affiliations:** 1 Institute of Psychology, University of Szczecin, Krakowska 69 Street, 71-017 Szczecin, Poland; sylwia.michalowska@usz.edu.pl; 2 Primary School with Bilingual and Kindergarten Sections, Wkrzańska 19a Street, 72-020 Trzebież, Poland; gnychalicja@gmail.com; 3 Alojzy Orione Primary School No. 5, Łaska 84 Street, 98-220 Zduńska Wola, Poland; albinarybarska139@gmail.com; 4 Psychotherapy Office Michał Brzegowy and Team, Śliczna 34B/101 Street, 31-444 Kraków, Poland; strochalskaklaudia@gmail.com

**Keywords:** temperament, self-harm, tendency to experience tension, perfectionism

## Abstract

**Background:** Adolescence is an important point in the emotional development of young people. It is a time when young people are characterised by a high degree of emotional instability and seek effective ways to regulate their emotions. One of the frequent methods they use to cope with emotional tension is self-injurious behaviour. **Methods:** In the context of the rising incidence of self-harm among adolescents, this study aims to understand the association of temperament with the experience of tension and self-injurious behaviour along with the mediating role of perfectionism among 366 adolescents aged 15 to 20 years (Mage = 17.98, SD = 1.302, 52.7% female). Participants completed questionnaires on temperament traits, level of perfectionism, and experience of tension and self-injurious behaviour. **Results:** The results show that traits such as perfectionism, sensory sensitivity and emotional reactivity increase the risk of self-injurious behaviour. Maladaptive perfectionism partially mediates the relationship between these traits and the tendency to experience emotional tension. A temperament profile with a protective role was also identified. **Conclusions:** The results of the study highlight the importance of innate traits as well as environmental and cognitive influences, and may contribute to a better understanding of the mechanisms leading to self-injurious behaviour and strategies aimed at its prevention.

## 1. Introduction

Adolescence is a time of intense transitions, including biological, social and emotional changes, as well as identity transitions, which increases the risk of developing mental health problems [1]. Self-harm, understood as socially unacceptable self-injurious behaviours that are intentional, undertaken of one’s own free will and not life-threatening, involving damage to one’s own body, [2], is quite common among adolescents [3] and serves a number of functions, such as emotion regulation, self-punishment, prevention of dissociation or regaining control [4]. Recent analyses also highlight the importance of perfectionism as a risk factor for self-injurious behaviour in this age group [5].

### 1.1. Regulative Theory of Temperament

Human temperament has been described in various contexts. The understanding adopted in the present study refers to Strelau’s Regulative Theory of Temperament, according to which temperament refers to formal behavioural traits and is seen as comprising basic, relatively fixed personality traits determined by innate neurobiochemical mechanisms [6,7,8,9]. Conceived in this way, temperament undergoes gradual changes over the course of life as influenced by maturation and the interactions occurring between the genotype and the environment of a particular person [10]. Temperament is a two-dimensional construct–energetic and temporal. The energetic level includes an individual’s activity, reactivity, sensory sensitivity and endurance, while the temporal dimension allows one to identify briskness, a tendency to react quickly, maintain a high pace of activity, or ease in changing reactions. This dimension also points to perseveration, i.e., the tendency to continue the behaviour even after the stimulation has ended [10]. The different configurations of the indicated traits allow one to distinguish four temperament categories in line with the Hippocrates–Galen theory [9]: (1) temperamental structure harmonised with high stimulation processing capabilities (sanguine); (2) temperamental structure harmonised with low stimulation processing capabilities (melancholic); (3) temperamental structure not harmonised with high stimulation processing capabilities (phlegmatic); and (4) temperamental structure not harmonised with low stimulation processing capabilities (choleric).

Temperamental factors are important for emotional self-regulation–the experience of tension and self-injurious behaviour in adolescents.

### 1.2. Self-Injurious Behaviour and the Experience of Tension by Adolescents—The Role of Temperament

The phenomenon of self-aggressive behavior among young people is considered a serious public health problem [11].

The definition of self-injurious behaviour adopted in this study refers to adolescents engaging in actions aimed at harming themselves, both physically and psychologically [12]. According to [13], based on the answers given by the subjects in the Tension Situations Questionnaire, coping strategies were divided into two groups–self-injurious behaviour and non-self-injurious behaviour. Direct and indirect forms of resorting to self-injury to reduce emotional tension were considered as self-injurious behaviour, according to the definition used. Direct strategies included harming oneself (by hitting and/or hurting oneself), while indirect strategies included imagining harming oneself (by hitting and/or hurting oneself). Self-injurious behaviour, understood as causing physical harm to oneself or imagining such an act in response to a stressful situation, leads to negative reinforcement. Self-injurious behaviour is followed by a reduction of unpleasant emotions, and this reinforces the vicious circle of maladaptive coping. Over time, such behaviour becomes a conditioned response to strong, difficult emotional stimuli [14].

For years, researchers have been looking for risk factors for self-harm among young people, looking for them both among social factors–family and peers, negative childhood experiences, as well as among individual characteristics of the individual. However, it turns out that self-aggressive behaviours are the result of many factors at the same time [15], and it is difficult to determine which is the strongest factor. The most important social factors that increase the risk of self-harm in young people most often include bullying by peers [16], social “contagion” from friends who self-harm or through observing self-harm on social media [17,18], exposure to social media [19] and domestic violence [20]. Individual factors include personality from cluster B and a specific temperament [21].

Recent research work addressing the topic of self-injury points to the importance of temperamental impulsivity as an important risk factor [22,23,24]. Researchers focusing attention on the relationship between temperament and self-harm have also demonstrated the importance of sensation-seeking tendencies by perceiving them as a predisposing factor [25]. The association of temperament, in particular novelty-seeking tendencies, harm avoidance and impulsiveness with self-harming behaviour in adolescents was also confirmed by the study of Tschan and colleagues [26].Meanwhile, possible neurological sources of self-harming behaviour linked to dysregulation of the opioid system, such as lower baseline levels of endogenous opioids, should not be neglected [27]. A currently developing line of research also aims to identify patterns of biological markers underlying nonsuicidal self-injury [28]. The findings of Case and colleagues [29] suggest a dysfunctional reward mechanism in self-injurious adolescents, even at the very early stage of displaying such behaviour. In the meta-analysis conducted by Favaretto et al. [30], affective temperament is a genetic factor underlying psychopathology, including mood disorders. According to the model proposed by Akiskal et al. [31], affective temperaments are defined as “specific modalities-which are stable and genetically determined-by which each individual experiences emotionality and affectivity” [30] (p. 407). They consist of five types of temperament: Hyperthymic, Cyclothymic, Depressive, Irritable and Anxious. According to the latest research, cyclothymic temperament, characterized by extreme affective and cognitive lability and unstable self-esteem, is a risk factor for the occurrence of, among others, suicidal behavior, BPD and severe depression. A relationship has also been shown between depressive and anxious temperament and the occurrence of emotional dysregulation [30].

Research based on the Regulative Theory of Temperament has shown that individuals who are more prone to taking risks are characterised by higher activity, endurance and briskness as well as lower reactivity [32], while adolescents prone to behavioural addictions are characterised by high perseveration and emotional reactivity as well as low briskness and endurance [33]. In other studies, the risk was specifically linked to emotional reactivity, briskness and sensory sensitivity [34]. It has also been pointed out that low sensory sensitivity may be critical to the degree of engaging in alcohol consumption [35]. The findings described may be relevant given that repeated self-injury is fixed through the experience of reward, in the form of reinforcement resulting from an increase in dopamine levels [36], or relief following an act of self-injury, and may resemble an addiction mechanism [37,38]. Anxiety, tension and agitation preceding self-injury are compared to withdrawal symptoms experienced by patients suffering from substance use disorders [39]. Individuals who engage in self-injurious behaviour experience urges and desires of a nature similar to those of substance addicts [40].

An important factor that determines the emergence of self-injurious behaviour in adolescents is their tendency to experience a certain level of tension in challenging situations. This tendency may also be temperamentally determined [41]. This work adopts [13] definition of tension, which is nominal and ostensive in nature, and is understood as ‘an unpleasant state, discomfort, feeling tense, nervous, unpleasantly aroused. It may sometimes be experienced in the body and may be associated with emotions or thoughts’ [13] (p. 57). Tension-inducing situations (based on analyses of blogs written by people who engage in self-injurious behaviour, literature and analyses of questionnaires measuring tension) include those in which the young person experiences failure, unpleasant emotions (towards self and/or others/the world) or frustration, feels a rush of thoughts and feelings, feels or actually is misunderstood or rejected, feels a lack of control, as well as situations of conflict with loved ones, social pressure, waiting for or while being judged, and while experiencing cognitive dissonance [13].

The results of research with adolescents have shown that there is a relationship between temperament traits, the experience of tension, the ability to regulate emotions and self-injurious behaviour [42]. Kubacka-Jasiecka [43] suggests the significance of biological predispositions, according to which an individual reacts quickly to stimuli with strong responses associated with intense emotions (experiencing high tension), which favours the fixation of self-injurious behaviour as a way of coping. When looking for the motivational factors behind young people’s self-injurious behaviour, attention is primarily drawn to difficulties in regulating emotions [44,45,46]. This is relevant to the analyses described in this paper, considering that the ability to regulate emotions in an effective manner is related to an individual’s temperament traits [47,48,49]. It appears that some of the functions of self-injurious behaviour, such as self-punishment or prevention of dissociation, may simultaneously reduce tension [50]. Self-injurious behaviours may also be solely focused on reducing the discomfort and tension felt by the young person. Analyses indicate that girls tend to self-harm for emotional reasons, while boys often do so for social reasons [51]. Difficulties in regulating emotions, including the inability to cope with emotional tension, are some of the most significant aspects concerning the aetiology of self-harm in adolescents [52]. Much of the research to date has shown that there is a link between recent stressful events (and therefore experiencing emotional tension) in adolescents‘ lives and adolescents’ engagement in nonsuicidal self-injury [20,53] and that this is linked to the severity of self-inflicted injuries [54]. However, in terms of the tension experienced, young people who self-injure are probably not different from those who do not self-injure, but show significantly greater difficulty in tolerating this emotional discomfort. Of particular relevance to the described relationship between the tendency to experience increased tension and the inability to regulate these sensations and self-injurious behaviour is the maladaptive form of perfectionism.

### 1.3. Maladaptive Perfectionism as an Important Link in the Relationship Between Temperament and Feelings of Tension and Self-Injury in Adolescents

Not all individuals with certain temperamental tendencies engage in self-injurious behaviour. Despite biological predispositions linked to an increased tendency to experience tension, young people adopt different coping strategies. One of these is increased control that may be reflected in maladaptive perfectionism. In 1990, Frost constructed a concept of perfectionism based on six components, such as attaching high importance to mistakes, high personal standards, doubts about actions taken, need for good organisation, high parental expectations and high parental criticism [55]. Based on the factors thus identified, two types of perfectionism correlated with the experience of positive or negative emotional states have been distinguished [56], leading to a distinction between adaptive perfectionism (otherwise known as striving for perfection) and maladaptive perfectionism (known as perfectionistic anxiety) [57,58]. Of relevance here is the dimensional approach to understanding perfectionism that has been explored for years. Hewitt and Flett [59] distinguished between self-oriented perfectionism (SOP), referring to the demands an individual imposes on him or herself in pursuit of perfection, and socially prescribed perfectionism (SPP), concerning the external demands associated with an individual’s perception of the environment as one that expects perfection.

Referring to the relationship between a person’s temperament and the perfectionism they exhibit, temperamental factors have been shown to predict individual dimensions of perfectionism [60]. Some authors have pointed out that maladaptive and adaptive perfectionism are related to distinct temperamental characteristics [60]. Research analyses published in 2023 [61] focusing on temperament and perfectionism profiles in individuals with high ability have highlighted that it is temperament that may play an important role in explaining the heterogeneity observed in groups of individuals with perfectionist traits. It is therefore very important to consider not only the dimension of perfectionism, but also its aetiology [62].

Research has shown that perfectionism is associated with emotional dysregulation, increasing the risk of engaging in maladaptive coping strategies. It has been indicated that emotional dysregulation in individuals displaying maladaptive perfectionism is rooted in increased negative affect occurring in response to threatened high standards, with implicit and explicit maladaptive emotion regulation strategies contributing to further dysregulation and increased negative affect [63]. The literature also describes a model of perfectionism as a predictor, whereby displaying such traits can predict how people regulate emotions in stressful situations and how this affects their mental health [64]. Research with adolescents has shown that self-oriented perfectionism is more often associated with the use of adaptive emotion regulation strategies, whereas socially imposed perfectionism predicts an increase in difficulties in regulating emotions, including difficulty in accepting the experienced states, and–of importance from the context of the research undertaken–difficulty in controlling impulsive behaviour [65]. A systematic research analysis published in 2021 [5] found a link between perfectionism and self-injurious behaviour without suicidal intentions, suggesting the inclusion of perfectionism in proposed prevention activities. Later studies also confirmed the significant importance of perfectionism in the aspects indicated [66]. Analyses have also shown that elevated levels of perfectionism, and the associated lower self-esteem, can lead to self-injurious behaviour [67]. Maladaptive perfectionism, associated with high personal standards and a greater tendency toward stress and self-injurious behavior, is well-documented in the literature [66,68]. At the same time, recent data suggest that its severity in adolescents is increasing [69], intensifying the efforts of both researchers and practitioners to understand the described mechanisms and also to develop and implement appropriate action strategies.

### 1.4. Current Study

Based on theoretical sources and empirical evidence, a number of research hypotheses were formulated as the basis for the analyses undertaken in this study. It was assumed that there is a relationship between temperament and perfectionism and a tendency to experience tension. It was hypothesised that higher levels of perseveration and emotional reactivity were associated with maladaptive perfectionism, and that higher emotional reactivity, perseveration and lower endurance correlated with a tendency to experience more severe tension. The authors also hypothesised that there is a relationship between maladaptive perfectionism and the tendency to experience tension, and that the higher the tendency, the higher the severity of maladaptive perfectionism. Based on previous knowledge, it was hypothesised that there are differences between temperament profiles in their relationships with maladaptive perfectionism, the tendency to experience tension, and self-injurious behaviour, and that a temperament characterised by higher perseveration, sensory sensitivity, emotional reactivity and lower endurance, compared to other profiles, is associated with maladaptive perfectionism, the tendency to experience tension, and self-injurious behaviour. It was hypothesised that the mechanism of maladaptive perfectionism plays an important mediating role in the association between the temperament profile predisposing to self-injurious behaviour and the tendency to experience tension, and that maladaptive perfectionism intensifies the association between temperament predisposing to self-injurious behaviour and the tendency to experience tension.

## 2. Materials and Methods

### 2.1. Participants

A total of 366 Caucasian subjects, from six secondary schools in north-western Poland, aged between 15 and 20 years (M = 17.98, SD = 1.302) participated in the study. Due to missing or erroneous data in the questionnaire packets received from the subjects, 338 test batteries were used for the analyses. The sample included 52.7% girls (178 subjects), 44.1% boys (149 subjects), and 3.3% (11 individuals) who did not identify with a binary gender.

Nineteen percent of the students attended the first year of secondary school, 18% attended the second year,16% attended the third year, and 47% of the students attended the fourth year. A total of 229 students (67.8%) attended the best secondary schools in Szczecin, awarded the Golden Shield in the Perspektywy ranking. The Perspektywy ranking is a Poland-wide ranking that selects the best 1000 institutions from among all Polish secondary schools and technical schools. The first 200 are awarded the Golden Shield. Points are awarded for success in science Olympiads and results from secondary school final exams.

Among the study participants, 23.56% were residents of a small town (up to 20,000 inhabitants), 10.63% of a medium-sized town (from 20,000 to 100,000 inhabitants), and 65.80% of a town with more than 100,000 inhabitants.

### 2.2. Procedure

The study was approved by the Bioethical Committee No. KB17/2023. The project was conducted in secondary schools in which the principals, students and their legal guardians gave written consent to participate in the study. The selection of teenagers for the study group was based on convenience sampling. In order to maintain the representativity of the sample, the people selected for the study were of mixed gender, purposefully selecting secondary schools with different levels of education, while the selection of classes and students was random.

The study took place during lessons, with the consent of the teacher in charge and with the supervision of the researcher. Students completed the questionnaires in 40–50 min. They received a link to the study via the internet and completed questionnaires created beforehand on the Google Forms platform on their phones or school computers. Participation in the study was voluntary, and all participants remained anonymous. Before starting the study, participants were read the instructions and informed of the purpose of the study and their right to opt out at any time during the study.

### 2.3. Measures

The following research tools were used in the study: a self-administered questionnaire, the Tension Situations Questionnaire by Kubiak [13], The Polish Adaptive and Maladaptive Perfectionism Questionnaire (KPAD) [57] assessing perfectionism, and the Formal Characteristics of Behaviour-Temperament Questionnaire Revised [70].

### 2.4. Perceived Tension and Self-Injurious Behaviour

The Tension Situations Questionnaire (KSN) [13] was used to assess the strength of the tension experienced by adolescents in various difficult situations and the tendency to react to them in an adaptive or self-injurious manner. The KSN questionnaire consists of 20 items describing situations that may cause tension. The respondent must determine how tense they are in a given situation by marking the answer on a scale from 0 to 10, where 0 means no tension and 10, very strong tension. Then, in each item, they are asked to identify the behaviour they exhibit in the described situation by marking as many behaviours as they want from a list of 13 possible. They can also write in a behaviour that is not listed. The answers are then classified into one of two categories: self-harming behaviours or non-self-harming behaviours. The original reliability of the questionnaire items ranged from 0.69 to 0.99. Cronbach’s alpha in the self-administered study was 0.93.

### 2.5. Adaptive and Maladaptive Perfectionism

Another variable analysed in the study was perfectionism. The Polish Adaptive and Maladaptive Perfectionism Questionnaire (KPAD) [57] was used to assess it. The KPAD was based on Frost’s [55] theory of perfectionism and consists of 35 items belonging to two scales: Adaptive Perfectionism and Maladaptive Perfectionism. The respondent provides a response on a 7-point Likert scale (1—strongly disagree, 7—strongly agree). The higher the score on each subscale, the greater the perfectionism of a given type. The original reliability for the Maladaptive Perfectionism scale was α = 0.953 and for the Adaptive Perfectionism scale, α = 0.896. Cronbach’s alpha in the self-administered study was 0.896 for the Adaptive Perfectionism scale and 0.952 for the Maladaptive Perfectionism scale.

### 2.6. Temperament Dimensions

Indicators of the adolescents’ temperament variable were the scores obtained by the subjects on the Formal Characteristics of Behaviour-Temperament Questionnaire Revised (FCZ-KT (R)). The FCZ-KT(R) is used to diagnose the basic, physiologically determined temperamental dimensions that are a component of personality according to the Regulative Theory of Temperament [10]. The questionnaire consists of 100 items to which the subject has to respond on a four-point scale, where 1 means strongly disagree and 4 means strongly agree. The results cover seven scales. There are 15 items in each scale, except for the Rhythmicity scale, which has 10 items. The first three, namely Briskness, Perseveration and Rhythmicity, belong to the temporal characteristics of behaviour, and the remaining four, namely Sensory Sensitivity, Endurance, Emotional Reactivity and Activity, belong to the energetic level of behaviour.

The high reliability of the questionnaire is evidenced by internal consistency coefficients ranging from 0.73 to 0.88 in the validation sample, and the high temporal stability of the results is evidenced by high correlation coefficients between the scales, ranging from 0.76 to 0.90.

In the study conducted by the authors, reliability measures were also determined for the individual scales. Their values remained similar to those obtained in the validation sample. Higher reliability was obtained for the endurance (α = 0.824), rhythmicity (α = 0.854), activity (α = 0.893), sensory sensitivity (α = 0.83), perseveration (α = 0.824) and briskness (α = 0.797) scales. The same level of reliability was obtained for the emotional reactivity scale (α = 0.881).

### 2.7. Sociodemographic Variables

The self-administered questionnaire contained questions that allowed the collection of basic sociodemographic data about the adolescents studied, such as age, gender identity, type and number of secondary schools attended, educational level (grade) and place of residence.

### 2.8. Strategy of Analyses

Statistical analyses were carried out using IBM SPSS Statistics v. 29 and Jamovi 2.4.11. Using the programme, basic descriptive statistics were calculated together with testing for normality of the distribution (Kolmogorov–Smirnov test). In the next step, a Pearson correlation (r) analysis was conducted to establish relationships between the dimensions of personality, perfectionism and the tendency to experience tension. This was followed by an analysis of latent profiles for the temperament dimensions. The highlighted profiles were compared in terms of the analysed variables using a one-way analysis of variance. Tukey’s HSD test was used as a post hoc test.

In order to compare the profiles in terms of engaging in self-injurious behaviour, an analysis was performed using the χ^2^ test of independence.

In a final step, using the PROCESS macro of A. Hayes (model 4), the mediating role of maladaptive perfectionism was tested for the relationship between the profile predisposing to self-injurious behaviour and the tendency to experience tension. α = 0.05 was used as the level of significance.

## 3. Results

### 3.1. Descriptive Statistics

Table 1 presents the basic measures of descriptive statistics together with the Kolmogorov–Smirnov test of normality of the distribution. The analysis showed a distribution consistent with a normal distribution for endurance, activity and maladaptive perfectionism. For the remaining variables, the distribution deviated from the normal distribution, with skewness values for all of these variables falling within the <−1;1> range, meaning that the distribution deviated slightly from the normal distribution [71].

### 3.2. Correlations Between Temperament Dimensions, Perfectionism and Tendency to Experience Tension

Table 2 presents the correlation matrix of the analysed variables. A negative and weak relationship was found between maladaptive perfectionism and briskness, endurance and activity–higher levels of perfectionism were associated with lower levels of these temperament dimensions. A strong and positive relationship was found between perfectionism and perseveration–with higher levels of maladaptive perfectionism, higher levels of perseveration were found. Adaptive perfectionism was positively and weakly correlated with briskness, rhythmicity, sensory sensitivity and activity. Higher levels of tendency to experience tension were associated with lower levels of briskness (negative, weak correlation) and endurance (negative, moderate correlation) and with higher levels of perseveration, emotional reactivity and maladaptive perfectionism (positive, strong correlations).

### 3.3. Latent Profiles Based on Temperament Traits

Based on the analysis of the latent profiles, a decision was made to separate four groups of subjects (Entropy = 0.761; AIC = 0.6468, Table 3).

### 3.4. Characteristics of Temperament Profiles

Table 4 presents the results of the one-way analysis of variance comparing the separate groups of subjects.

Briskness. The analysis showed that individuals in profiles 1 and 4 manifested similar levels of briskness (*p* = 0.959) and this was significantly lower than in profile 3 (*p* < 0.001) and significantly higher than in profile 2 (*p* ≤ 0.048).

Rhythmicity. A higher level of rhythmicity was manifested by respondents from group 4 than from group 1 (*p* < 0.001). No differences were noted between the other groups.

Perseveration. For perseveration, differences were noted between all compared groups of subjects (*p* < 0.001). The highest level of this trait was recorded in profile 2, followed by profiles 1 and 3, and the lowest level of perseveration for profile 4.

Sensory sensitivity. There were no significant differences in the intensity of sensory sensitivity between subjects from groups 2 and 3 (*p* = 0.363). Respondents from the other two profiles obtained significantly lower scores for this trait than respondents from groups 2 and 3 (*p* < 0.001).

Endurance. Significant differences in endurance intensity were found between all profiles (*p* ≤ 0.006). Subjects from group 3 manifested the highest endurance, followed by subjects from groups 4 and 1, and the lowest level of endurance occurred among subjects from group 2.

Emotional reactivity. For reactivity, significant differences were also noted between all profiles. The highest level of reactivity occurred in profile 2, followed by profile 1 and then group 4 and group 3 (*p* < 0.001).

Activity. Group 3 was found to have significantly higher levels of activity than groups 1 (*p* < 0.001), 2 (*p* = 0.006) and 4 (*p* < 0.001), with no differences in the intensity of this trait between the other groups (*p* > 0.05).

**Figure 1:**
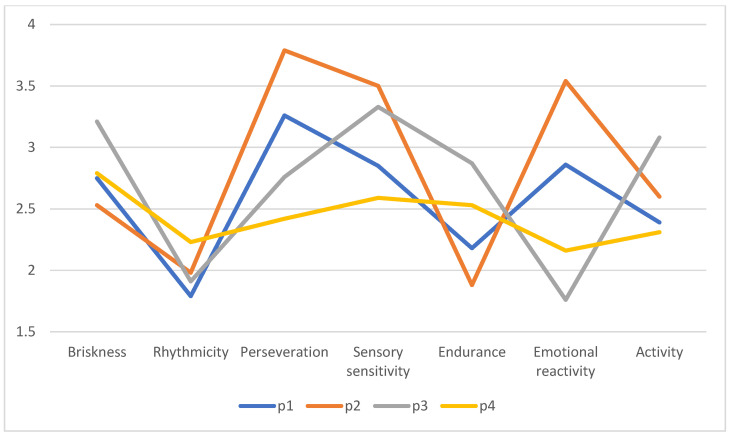
Average intensity of temperament traits according to latent profiles. Source: own research.

Figure 1 show Average intensity of temperament traits according to latent profiles.

### 3.5. Comparison of Temperament Profiles in Terms of Perfectionism Dimensions and Tendency to Experience Tension

In order to compare the distinguished profiles in terms of perfectionism dimensions and the tendency to experience tension, a one-way analysis of variance was conducted. The analysis showed significant differences between groups for all analysed variables (Table 5).

Maladaptive perfectionism. There were no significant differences in the intensity of maladaptive perfectionism between those in groups 3 and 4 (*p* = 0.124). In profile 2, the level of tension was significantly higher than in the other groups (*p* < 0.001), and in group 1, it was higher than in groups 3 and 4 (*p* < 0.001).

Adaptive perfectionism. In profile 1, the level of adaptive perfectionism was significantly lower than in profile 2 (*p* = 0.028) and profile 3 (*p* < 0.001). In profile 3, the intensity of perfectionism was higher than in profile 4 (*p* < 0.001). There were no differences between the other groups (*p* > 0.05).

Tendency to experience tension. There were no significant differences in the severity of the tendency to experience tension between individuals in profile 3 and profile 4 (*p* = 0.432). In profile 2, the level of tension was significantly higher than in the other groups (*p* < 0.001), and in group 1, it was higher than in groups 3 and 4 (*p* < 0.001).

### 3.6. Temperament Profiles and Engaging in Self-Injurious Behaviour

On the basis of the KNS questionnaire, the respondents were divided into two groups: those who engage in self-injurious behaviour (‘I hurt myself’ was indicated at least once in the answers) and those who do not. These groups were then compared in terms of prevalence in each profile using a χ^2^ test of independence. The analysis showed a significant association between the variables, χ^2^(3) = 24.37; *p* < 0.001; *V* = 0.27. Profile 2 respondents engaged in self-injurious behaviour most frequently, while Profile 3 showed the lowest frequency. The frequency analysis is shown in Table 6.

In summary, profile 2 featured individuals with elevated levels of perseveration, sensory sensitivity and emotional reactivity, and reduced endurance and briskness. This profile was also characterised by elevated levels of perfectionism and a higher tendency to experience tension. Respondents in this profile also showed a greater tendency to engage in self-injurious behaviour than those in the other profiles. Consequently, this profile was included in further analysis as predisposing to self-injurious behaviour.

### 3.7. The Mediating Role of Maladaptive Perfectionism in the Relationship Between Temperament Predisposing to Self-Injurious Behaviour and the Tendency to Experience Tension

To determine whether maladaptive perfectionism was a significant mediator of the relationship between temperament predisposing to self-injurious behaviour and the tendency to experience tension, a mediation analysis was conducted using the macro PROCESS of A. Hayes (model 4).

**Figure 2:**
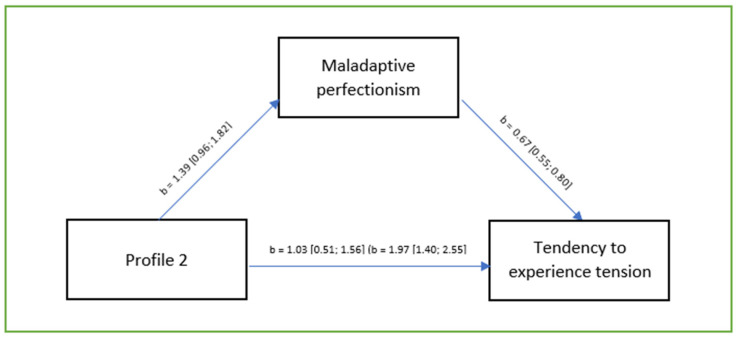
Unstandardised regression coefficients for the mediation model. Annotation. Regression coefficients for pathway c are given in brackets. Source: own research.

The model analysed was a good fit to the data. *F* (1.336) = 39.77; *p* < 0.001; R^2^ = 0.11. There was a positive correlation between the presence of a temperament predisposing to self-injurious behaviour and maladaptive perfectionism (pathway a: *b* = 1.39; *p* < 0.001; 95% CI [0.96; 1.82]). Perfectionism was higher among profile 2 respondents. The correlation of maladaptive perfectionism with the tendency to experience tension (when controlling the temperament profile) was positive (pathway b: *b* = 0.67; *p* < 0.001; 95% CI [0.55; 0.80]). Higher levels of maladaptive perfectionism were associated with a higher tendency to experience tension. The correlation between temperament predisposing to self-injurious behaviour and tendency to experience tension was also positive (pathway c: *b* = 1.97; *p* < 0.001; 95% CI [1.40; 2.55]) and also remained significant after including perfectionism in the model (pathway c’: *b* = 1.03; *p* < 0.001; 95% CI [0.51; 1.56]). The significance of the indirect effect was estimated using the bootstrapping method for sampling of 5000. Analysis revealed a significant indirect effect of perfectionism for the relationship between temperament predisposing to self-injurious behaviour and the tendency to experience tension (*b* = 0.94; 95% CI [0.36; 1.24]). This effect indicates the presence of partial mediation (Figure 2).

## 4. Discussion

Adolescence is an important point in the emotional development of young people. It is a time when young people are characterised by a high degree of emotional instability and seek effective ways to regulate their emotions. One of the frequent methods they use to cope with emotional tension is self-injurious behaviour. The frequent use of these strategies is a result of not yet developed self-regulation skills and immature behavioural mechanisms [52]. According to previous reports, the intensity of emotional tension experienced by young people, and how it is reduced, depends on biopsychosocial factors [72].

The second half of adolescence is associated with intensive learning in secondary schools. This is a time of social comparison, building one’s independence and identity [73]. An important mechanism that determines the quality of an adolescent’s emotional functioning and strongly influences his or her assessment of and response to reality is perfectionism [62]. Perfectionism causes a young person to feel pressured in some way to meet very high standards that affect how they later think about themselves. Some perfectionism is helpful—it builds motivation to act and allows them to achieve accomplishments; the other part is maladaptive, often leading to the development of developmental psychopathology [57,58]. Research shows that maladaptive perfectionism in adolescence is associated with self-injurious behaviour [5,67]. These behaviours most likely serve as a tool to reduce the immense tension caused by the inability to achieve very high standards set for oneself, feeling a sense of constant failure and disappointment with oneself [14,66].

The study presented here aimed to determine the mediating role of maladaptive perfectionism occurring in adolescents, in the relationship between their temperament profile, which is most closely associated with a tendency to engage in self-injurious behaviour, and the severity of their feelings of emotional tension in various situations triggering this emotion. The results obtained in the present study confirmed, in whole or in part, all the hypotheses raised.

### 4.1. Relationships Between Temperament Dimensions and Perfectionism and Tendency to Experience Tension

The results reveal that adolescents characterised by greater temperamentally conditioned emotional instability (higher levels of perseveration) have a greater tendency to exhibit maladaptive perfectionism. Other temperament traits have little association with perfectionism, whether adaptive or maladaptive. According to Strelau’s [10] theory, adolescents characterised by high perseveration tend to repeat reactions, behaviours or words, and to over-analyse previous events or decisions. Consequently, they relive emotions for a very long time and focus on feelings experienced in the past, are very sensitive and may experience increased tension in subjectively neutral situations. This implies that they may display a tendency towards maladaptive perfectionism, as they focus on failure and the associated negative emotions, and in order to control these emotions, they may take further actions aimed at proving to themselves that they are able to meet the imposed requirements or standards. The results are consistent with other reports where temperamental factors, particularly an anxious or inhibited temperament, may increase the likelihood of developing a dimension of maladaptive perfectionism. An anxious temperament may increase the level of maladaptive perfectionism by increasing the perception of threat in the form of new or difficult situations and the fear of possible distress experienced in these situations [60].

The following analyses establish the existence of a relationship between temperament and the experience of emotional tension by the adolescents studied. As expected, the traits associated with intense emotionality and anxiety, i.e., higher emotional reactivity, perseveration, but also lower endurance, are again linked to a tendency to experience stronger tension in different situations. The results obtained are in line with the theoretical assumptions of temperament by Strelau [10], where, in addition to perseveration, emotional reactivity, understood as the intensity of the emotional response to various stimuli, is particularly important for experiencing increased tension. According to the theory, highly reactive people are distrustful and avoid difficult relationships and events. They often withdraw and experience very strong emotional tension, even in objectively neutral situations [10]. The authors of this article did not find other analyses that explicitly show the relationships studied. The results obtained are, however, reflected in the results of related studies in which the tendency to experience negative emotions, including chronic tension, has been linked to neuroticism [74]. A 2022 study of college students found that neuroticism can influence engaging in self-injurious behaviour without suicidal tendencies [75]. Young people’s feelings of tension and the search for relief through self-harm are linked to the inability to regulate emotions on the one hand and the intensity of the emotional states experienced on the other. Consequently, self-harm can be used to increase affective experiences (feeling more) and to reduce them (feeling less) [72].

The results obtained by the authors reveal the existence of a relationship between adolescents’ maladaptive perfectionism and their tendency to experience tension. They found that adolescents characterised by maladaptive perfectionism also experience stronger tension. Maladaptive perfectionism creates persistent tension, even in situations others perceive as non-threatening. On the other hand, the tendency to feel strong emotional tension in various situations may trigger the mechanism of maladaptive perfectionism in adolescents to reduce this tension and regulate emotions. The result obtained is in line with previous research, where perfectionism was associated with poorer acceptance of experienced states, lower self-esteem and a tendency to feel increased tension [66,67]. These results are also consistent with the analyses of Sand and colleagues [76], in which temperament related to emotional regulation and social pressures often led to higher levels of maladaptive perfectionism, which is linked to issues like anxiety and depression in adolescents. Another study focused on gifted adolescents revealed that perfectionism in this group could be categorized into different types based on their temperamental and personality profiles. It showed that a “Pervasive” perfectionism type, which included both adaptive and maladaptive tendencies, was particularly prominent in individuals with a high need for achievement, a common feature in certain temperamental profiles. This study also highlighted that perfectionism could be influenced by both personal and environmental factors, such as pressure from parents and academic expectations [77].

### 4.2. Relationship of Temperament Profiles to Perfectionism, Experience of Tension and Self-Injurious Behaviour

Subsequent statistical analyses allowed us to distinguish four temperament profiles, two of which play a particular role in the relationship with perfectionism, experienced tension and self-injurious behaviour in adolescents.

Profile 2, which differs from the others in higher perseveration, sensory sensitivity, emotional reactivity and lower endurance, is concurrently associated with maladaptive perfectionism, a tendency to experience tension and engage in self-injurious behaviour. Remarkably, it is also associated with adaptive perfectionism, more so than the other profiles (but similarly to profile 3). This means that the studied adolescents characterised by this profile tend to manifest both types of perfectionism. Previous research in this area has indicated that temperament traits can have a significant impact on whether perfectionism in adolescents is adaptive or maladaptive. Extraversion, conscientiousness and emotional stability are often associated with adaptive perfectionism, whereas neuroticism and high emotionality may lead to maladaptive perfectionism [78,79]. Results from other studies have also shown that temperament traits such as high emotional reactivity, impulsivity and low endurance may contribute to an increased risk of experiencing emotional tension and engaging in self-injurious behaviour in adolescents [80,81]. A study by Boyes et al. [82] examined the impact of temperament on the experience of emotional tension and self-injurious behaviour in adolescents. The results showed that temperament with high levels of neuroticism and low levels of emotional stability (synonymous in this study with high emotional reactivity and perseveration, with low endurance) was correlated with higher tension and more frequent self-injurious behaviour. A study by Tatnell et al. [83] analysed the relationship between temperament, experience of stressful life events and self-injurious behaviour in adolescents. The results showed that adolescents with temperament with high levels of emotional reactivity were more likely to engage in self-harm in response to stressful life events.

In the current study, adolescents with temperament profile 3 were characterised by the most harmonised structure, indicating a high capacity to process stimulation and high adaptability. Profile 3 is, by its structure, most similar of all to the sanguine type [84].Adolescents with this profile are characterised by good briskness (i.e., the ability to move smoothly and efficiently from one activity to another, a good pace of action), rhythmicity (e.g., in the circadian rhythm), average sensitivity, high activity (social-educational) and reduced perseveration and emotional reactivity. This profile is most weakly associated, compared to the rest of the profiles, with maladaptive perfectionism, a tendency to experience severe tension and engage in self-injurious behaviour, while, together with profile 2, it is most strongly associated with adaptive perfectionism, confirming the strong adaptability of individuals characterised by this type of temperament, as indicated by many previous studies [85]. These studies revealed that traits characteristic of sanguine people, such as optimism, sociability and flexibility, may contribute to better mental health, especially in the context of non-destructive ways of coping with stress and severe tension associated with difficult life situations. The authors of the article did not find studies that show a direct correlation between sanguine temperament and perfectionism, but there are some that may indirectly provide insight into this issue. For example, a study by Stoeber and Corr [85] found that extraversion, which is a hallmark of sanguine individuals, is negatively correlated with unhealthy forms of perfectionism. The results of a study by Hill and Curran [78] suggested that extraversion is positively correlated with adaptive perfectionism and life satisfaction. Although this research does not focus exclusively on the sanguine temperament, it does provide some clues about how the traits of this temperament type can help people cope with imposed standards in a healthy way.

The results obtained also confirm the drastic statistics indicating that between 16 and 19% of young people engage in self-injurious behaviour e.g., WHO [86], CDC, [87]. More than 20% of adolescents in temperament profiles 1 and 4, at least once in their responses, pointed to engaging in self-injurious behaviour in response to experienced tension. As mentioned above, the lowest percentage of such individuals was characterised by temperament profile 3 (3% of individuals), but frighteningly, more than 51% of adolescents with temperament profile 2 indicated engaging in this type of behaviour. Temperament profile 2 can be considered as predisposing to self-injurious behaviour.

### 4.3. Maladaptive Perfectionism in the Relationship Between Temperament Predisposing to Self-Injurious Behaviour and the Tendency to Experience Tension

The authors of this article made the assumption that the mechanism of maladaptive perfectionism plays an important mediating role in the relationship between the temperament profile predisposing to self-injurious behaviour of adolescents and their tendency to experience severe tension. In line with previous research, it was predicted that maladaptive perfectionism would intensify this relationship. Research has consistently shown that perfectionism in adolescents is associated with increased emotional tension. Adolescents with maladaptive perfectionism tend to experience heightened emotional distress, which often manifests as anxiety, anger, shame, and a deep sense of failure when they do not meet their self-imposed high standards. Such emotional reactions are compounded by a critical mindset and an inability to tolerate mistakes, which contributes to psychological strain and can even lead to burnout [88]. The results obtained show a partial mediation of maladaptive perfectionism, meaning that the relationship between temperament profile 2 and the tendency to experience severe tension, when perfectionism is introduced into the analyses, is significantly weakened, although it can still be observed in the results obtained. Temperament predisposing to self-injurious behaviour is associated with a stronger experience of tension among adolescents, and maladaptive perfectionism partially explains the relationship between the two. The results are consistent with the biopsychosocial model for self-injury [2], in which environmental, cognitive, affective and behavioural aspects play an important role in the experience of tension, in addition to innate traits. Perfectionism is one of the factors associated with the aspects indicated.

Favaretto et al. [30] highlight that certain affective temperament traits, such as emotional lability, increased perseveration, and high emotional reactivity, may act as key predisposing factors for the development of affective disorders, anxiety disorders, and self-harming behaviours in both adolescence and adulthood. Favaretto et al. [30] indicate that temperament plays a crucial role in modulating emotional intensity and regulation methods, which serves as a foundation for understanding individual differences in responses to stress. Their analyses suggest that adolescents with high emotional reactivity, especially those with a tendency towards maladaptive perfectionism, experience pronounced difficulties in managing emotional tension. This supports the hypothesis that intense emotional experiences, combined with perfectionist demands, may exacerbate tension and lead to self-harming behaviours, which young people often use as a means of emotional regulation.

In the present study, it was observed that adolescent perseveration and high emotional reactivity were significantly associated with experiencing increased tension and a tendency towards maladaptive perfectionism, consistent with the conclusions of Favaretto et al. [30]. The authors suggest that these temperament traits, particularly when combined with a lack of adequate emotional regulation mechanisms, may contribute to adaptive difficulties that frequently lead to the development of psychiatric disorders.

In their analysis, Favaretto and colleagues [30] emphasise the role of an “integrated model” that combines temperamental traits with social and cognitive factors in the development of self-harming behaviours. This model proposes that high emotional reactivity and a low level of adaptive coping strategies may significantly increase the propensity towards maladaptive perfectionism. In light of their analyses, these responses are reinforced by high levels of uncertainty and difficulties adapting to new situations—traits also exhibited by adolescents with temperament profile 2 in the study in question.

In conclusion, the present study confirms the important role of temperamental factors in perfectionism and the tendency to experience tension and self-injurious behaviour in adolescents. It also demonstrates a link between adolescents’ perfectionism and their experience of heightened tension. It reveals the existence of a temperament profile that plays a protective role for the development of psychopathology as a result of exhibiting maladaptive perfectionism, experiencing severe tension or engaging in self-injurious behaviour, and a profile predisposing to self-injurious behaviour that increases the perceived emotional tension. Finally, it shows that maladaptive perfectionism is only one factor explaining the relationship between temperament and the experience of tension by adolescents.

Referring to the practical implications of the presented results, several potentially important areas should be considered. Students with higher emotional reactivity may function better in an environment that allows for an individualised approach, permits a flexible work pace, and offers flexibility in assessment and emotional support. Workshops based on mindfulness and emotional regulation techniques could help build emotional resilience, facilitating coping with academic pressure. Cognitive-behavioural therapy techniques can support individuals with maladaptive perfectionism by helping them identify and change negative thought patterns that lead to excessive self-expectations. It may be worth incorporating these into school prevention programmes. High emotional reactivity and a tendency to experience tension point to the need for introducing emotional regulation techniques, such as mindfulness.

Temperament profile 2 represents an important starting point for developing effective early intervention protocols in the context of self-harm among adolescents. Individuals with a high level of perseveration tend to focus on negative thoughts and experiences, which can lead to chronic stress and anxiety. Combined with sensory sensitivity, which causes them to react more intensely to environmental stimuli (e.g., criticism, social pressure), this can result in stronger emotional responses such as anxiety or shame, which are often linked to self-destructive behaviours, as highlighted in the analyses by Gyori and Balazs [5]. Therefore, it seems that early interventions aimed at adolescents with temperament profile 2 could significantly reduce the risk of self-harm by improving the adaptive capacities of young people and supporting their mental health.

### 4.4. Limitations and Directions for Future Research

This study has several limitations. Firstly, it used a relatively small and specific sample of adolescents from a specific region. This limits the possibility to generalise the results to wider populations or other age groups. Secondly, much of the data was collected using self-administered instruments, which may be subject to errors such as a desire to present oneself in a better light or inaccurate self-assessment. This can affect the reliability and validity of the results obtained. Another limitation of the project may be the focus on specific factors–the study mainly focused on temperament and perfectionism in relation to self-injurious behaviour. Other potentially influential factors, such as environmental stressors, family relationships and genetic predisposition, were not thoroughly explored. A final limitation of the study is the lack of longitudinal data, which makes it impossible to track changes over time and to determine the long-term impact of temperament and perfectionism on self-injurious behaviour.

Future research should aim to include larger and more diverse samples from different demographic groups and geographical regions to increase the possibility of generalising the results. It would also be desirable to conduct longitudinal studies that could provide insights into the dynamic changes over time and the causal relationships between temperament, perfectionism and self-injurious behaviour. Future research should examine other factors contributing to the phenomenon, such as genetic influences, family dynamics, peer relationships and wider social contexts, in order to develop a more holistic understanding of self-injurious behaviour in young people. Finally, it would be worth noting the role of intervention research–analysing the effectiveness of targeted interventions aimed at reducing maladaptive perfectionism and improving emotional regulation could provide practical applications for preventing self-injurious behaviour among adolescents.

The identification and understanding of protective factors that reduce the risk of engaging in self-injurious behaviour despite high levels of perfectionism or specific temperament traits would also be valuable for the development of preventive strategies.

Given these limitations and research directions, future analyses may expand the results obtained, deepening our understanding of the complex interaction between temperament, perfectionism, and self-destructive behaviour in adolescents.

## 5. Conclusions

The results of the study highlight the important role of temperament and perfectionism in the context of the tendency to experience emotional tension and engage in self-injurious behaviour among adolescents. The analysis showed that there are different temperament profiles that can either protect against or predispose to self-injurious behaviour. Certain temperament traits, such as perseveration, sensory sensitivity and emotional reactivity, may increase the risk of self-injurious behaviour. Adolescents with a temperament profile characterised by these traits and low endurance and briskness are more likely to experience severe emotional tension, which in turn favours engaging in self-injurious behaviour. The results of the study also showed that maladaptive perfectionism plays a partially mediating role in the relationship between temperament predisposing to self-injurious behaviour and the tendency to experience tension. This means that adolescents with higher levels of maladaptive perfectionism are more likely to experience severe tension, which may lead to self-injurious behaviour.

The results are consistent with the biopsychosocial model of self-injurious behaviour, which takes into account innate traits of the individual as well as environmental, cognitive, affective and behavioural influences. Perfectionism, as an important cognitive factor, may contribute to increased feelings of tension and self-injurious behaviour. The identification of a temperament profile that plays a protective role against the development of psychopathology in the present study provides an opportunity to use this information for prevention and intervention. This profile, characterised by lower levels of maladaptive perfectionism and a lower tendency to experience tension, can provide a basis for designing support programmes for adolescents.

In conclusion, the study highlights the complexity of the relationship between temperament, perfectionism and self-injurious behaviour in adolescents. Its findings may contribute to a better understanding of the mechanisms leading to self-harm and to the development of more effective prevention and intervention strategies.

## Figures and Tables

**Table 1:** Descriptive statistics for the variables analysed together with the Kolmogorov–Smirnov test.

	M	Me	SD	Sk.	Kurt.	Min.	Max.	D	*p*
Briskness	2.78	2.80	0.51	−0.22	−0.10	1.07	3.93	0.05	0.029
Rhythmicity	1.90	1.80	0.64	0.53	−0.37	1.00	3.80	0.10	<0.001
Perseveration	3.12	3.20	0.50	−0.41	−0.16	1.47	4.00	0.07	<0.001
Sensory sensitivity	2.93	2.93	0.51	−0.20	0.00	1.40	4.00	0.06	0.004
Endurance	2.27	2.27	0.54	0.21	−0.23	1.07	3.80	0.05	0.063
Emotional reactivity	2.71	2.73	0.65	−0.26	−0.33	1.07	4.00	0.05	0.048
Activity	2.47	2.47	0.66	0.03	−0.68	1.07	3.93	0.04	0.200
Maladaptive perfectionism	4.29	4.27	1.40	−0.10	−0.75	1.00	7.00	0.04	0.200
Adaptive perfectionism	4.69	4.77	1.19	−0.25	−0.41	1.00	7.00	0.05	0.044
Tendency to experience tension	6.19	6.38	1.87	−0.49	−0.15	0.50	10.00	0.07	<0.001

Source: own research.

**Table 2:** Correlations between temperament dimensions, perfectionism and tendency to experience tension.

	1	2	3	4	5	6	7	8	9	10
1. Briskness	-									
2. Rhythmicity	0.04	-								
3. Perseveration	−0.11	−0.13 *	-							
4. Sensory sensitivity	0.05	−0.03	0.31 **	-						
5. Endurance	0.19 **	−0.03	−0.35 **	−0.04	-					
6. Emotional reactivity	−0.29 **	−0.05	0.61 **	0.08	−0.45 **	-				
7. Activity	0.18 **	−0.04	0.05	0.16 **	0.09	−0.15 **	-			
8. Maladaptive perfectionism	−0.27 **	0.02	0.51 **	0.06	−0.27 **	0.59 **	−0.15 **	-		
9. Adaptive perfectionism	0.19 **	0.22 **	0.07	0.13*	0.04	−0.08	0.17 **	0.05	-	
10. Tendency to experience tension	−0.18 **	−0.03	0.52 **	0.07	−0.36 **	0.59 **	−0.04	0.56 **	0.06	-

Annotation. * *p* < 0.05; ** *p* < 0.01. Source: own research.

**Table 3:** Comparison of fit measures of models with different numbers of latent profiles.

Class	Model	LogLik	AIC	AWE	BIC	CAIC	CLC	KIC	SABIC	ICL	Entropy
1	1	−3354	6735	6910	6789	6803	6709	6752	6745	−6789	1.000
2	1	−3246	6537	6813	6621	6643	6494	6562	6551	−6679	0.745
3	1	−3217	6493	6871	6608	6638	6435	6526	6513	−6710	0.725
4	1	−3196	6468	6947	6614	6652	6394	6509	6493	−6719	0.761

Source: own research.

**Table 4:** Comparison of latent profiles in terms of temperament dimensions.

Dependent Variable	1 (n = 204)		2 (n = 41)		3 (n = 33)		4 (n = 60)		F	df	*p*	η^2^
	M	SD	M	SD	M	SD	M	SD				
Briskness	2.75	0.49	2.53	0.54	3.21	0.30	2.79	0.52	22.72 ^a^	3; 94.29	<0.001	0.10
Rhythmicity	1.79	0.57	1.98	0.65	1.91	0.79	2.23	0.64	7.87	3; 334	<0.001	0.07
Perseveration	3.26	0.30	3.79	0.19	2.76	0.27	2.42	0.33	268.97 ^a^	3; 94.22	<0.001	0.66
Sensory sensitivity	2.85	0.46	3.50	0.34	3.33	0.35	2.59	0.42	64.11 ^a^	3; 95.21	<0.001	0.30
Endurance	2.18	0.46	1.88	0.49	2.87	0.52	2.53	0.48	35.02	3; 334	<0.001	0.24
Emotional reactivity	2.86	0.44	3.54	0.36	1.76	0.41	2.16	0.44	148.89	3; 334	<0.001	0.57
Activity	2.39	0.65	2.60	0.69	3.08	0.56	2.31	0.54	13.50	3; 334	<0.001	0.11

Annotation. ^a^ Welch’s correction; Source: own research.

**Table 5:** Comparison of profiles in terms of perfectionism and tendency to experience tension.

Dependent Variable	1 (n = 204)		2 (n = 41)		3 (n = 33)		4 (n = 60)		F	df	*p*	η^2^
	M	SD	M	SD	M	SD	M	SD				
Maladaptive perfectionism	4.49	1.25	5.51	1.00	2.94	0.98	3.52	1.34	37.54	3; 334	<0.001	0.25
Adaptive perfectionism	4.53	1.12	5.08	1.17	5.51	1.12	4.51	1.25	8.89	3; 334	<0.001	0.07
Tendency to experience tension	6.45	1.54	7.92	1.21	4.50	1.54	5.03	2.06	46.77 ^a^	3; 89.32	<0.001	0.26

Annotation. ^a^ Welch’s correction; Source: own research.

**Table 6:** Frequency analysis of engaging in self-injurious behaviour according to the temperament profile of the subjects.

	Profile 1		Profile 2		Profile 3		Profile 4	
	n	%	n	%	n	%	n	%
Not engaging in self-injurious behaviour	156 ^a^	76.5	20 ^b^	48.8	32 ^c^	97.0	47 ^a,c^	78.3
Engaging in self-injurious behaviour	48 ^a^	23.5	21 ^b^	51.2	1 ^c^	3.0	13 ^a,c^	21.7

Annotation. Any other letter in the superscript next to the values points to the presence of differences between the proportions at the *p* < 0.05 level (Bonferroni correction). Source: own research.

## Data Availability

The raw data supporting the conclusions of this article will be made available by the authors on request due to the sensitivity of the data collected during the study.
